# A Single Aptamer-Dependent Sandwich-Type Biosensor for the Colorimetric Detection of Cancer Cells via Direct Coordinately Binding of Bare Bimetallic Metal–Organic Framework-Based Nanozymes

**DOI:** 10.3390/bios13020225

**Published:** 2023-02-03

**Authors:** Yuhui Zhu, Xueting Fang, Xiaofei Lv, Meijun Lu, Hui Xu, Shengqiang Hu, Shulin Zhao, Fanggui Ye

**Affiliations:** 1 State Key Laboratory for the Chemistry and Molecular Engineering of Medicinal Resources, School of Chemistry and Pharmaceutical Sciences, Guangxi Normal University, Guilin 541004, China; 2 Nanxian Inspection and Testing Center of Yiyang City in Hunan Province, Yiyang 413299, China

**Keywords:** metal–organic frameworks, sandwich-type, single aptamer, coordination bond, colorimetric detection

## Abstract

A typical colorimetric sandwich-type sensor relies on dual antibodies/aptamers to specifically visualize the targets. The requirement of dual antibodies/aptamers and low signal intensity inevitably increases the design difficulty and compromises the sensing sensitivity. In this work, a novel sandwich-type aptasensor was developed using single aptamer-functionalized magnetic nanoparticles as a specific recognition unit to target cancer cells and a bimetallic metal–organic frameworks (MOFs)-based nanozymes as a colorimetric signal amplification unit. The well-defined crystalline structure of UIO-66 MOFs enabled the introduction of Fe/Zr bimetal nodes, which possessed integrated properties of the peroxidase-like nanozyme activity and direct coordinately binding to the cell surface. Such a novel construction strategy of sandwich-type aptasensors achieved simple, sensitive, and specific detection of the target cancer cells, which will inspire the development of biosensors.

## 1. Introduction

Cancer remains a major threaten to human life in the world [1]. Current clinical practice suggests that early detection of cancer cells contributes to monitoring the progressions of cancer and conducting precise therapy [2]. Consequently, it is desirable to develop simple, sensitive, and specific technologies to quantify cancer cells. Among all the developed detection strategies, colorimetric sandwich-type methods have attracted considerable attention due to the advantages of high specificity, easy operation, as well as bare-eye-based readout [3]. In general, the colorimetric sandwich-type assay is carried out by using two specific antibodies/aptamers to recognize the targets and using appropriate labels to provide detectable signals [4,5]. However, the requirement of two antibodies/aptamers inevitably increases the design difficulty. In fact, the use of single high-affinity antibody/aptamer fully meets the need of specific recognition of target cells [6,7,8]. In addition, low colorimetric signals greatly compromise the response sensitivity of visual analysis.

Metal–organic frameworks (MOFs) are a type of highly ordered porous crystalline material constructed by reasonable self-assembly of metal ions and organic ligands [9,10]. The well-defined crystalline structure of MOFs offers an excellent alternative to design metal nodes to acquire metal-related physicochemical properties, which holds great potential in colorimetric sandwich-type analysis. For instance, the introduction of Fe atoms might endow the resulting Fe-containing MOFs with intrinsic peroxidase-like catalytic activity [11], which could significantly increase the colorimetric analysis sensitivity via accelerating H_2_O_2_-mediated oxidation of chromogenic substrates. The coordination affinity between Zr^4+^ and PO_4_^3−^ contributes to identifying the cellular phospholipid bilayer using Zr-containing MOFs [12]. Correspondingly, developing bimetallic MOFs with a given ionic ratio offers an interesting candidate to obtain integrated properties from different metal nodes.

Herein, a novel sandwich-type aptasensor was prepared for colorimetric analysis of cancer cells using single aptamer-modified magnetic nanoparticles to selectively capture and separate target cells, followed by direct binding of bare bimetallic metal–organic-framework-based nanozymes. By adjusting the added amount of metal precursors among the synthesis of a given UIO-66 MOF, UIO-66(Fe/Zr) bimetallic MOFs were prepared with integrated properties, including the peroxidase-like nanozyme activity from Fe nodes and direct binding to cell surface based on the Zr-involved coordination bond. Such a single aptamer-dependent colorimetric sandwich-type aptasensor facilitated simple, sensitive, and specific detection of target cancer cells even in complex biological systems.

## 2. Experimental Section

### 2.1. Materials

Ferric chloride hexahydrate (FeCl_3_·6H_2_O) was purchased from Shanghai Yien Chemical Technology Co., Ltd. (Shanghai, China). Zirconium tetrachloride (ZrCl_4_), ferric oxide nanoparticles (Fe_3_O_4_ NPs), and (1-(3-dimethylamino-propyl)-3-ethylcarbon diamide hydrochloride (EDC) were obtained from Aladdin Reagent Co., Ltd. (Shanghai, China). Ethyl orthosilicate (TEOS) was purchased from Sinopharm Chemical Reagent Co., Ltd (Shanghai, China); 3-Aminopropyltriethoxysilane (APTES) and N, N-dimethylformamide (DMF) were attained from Titan Technology Co., Ltd. (Shanghai, China). Hydrogen peroxide (H_2_O_2_, 30 wt%), acetic acid, ammonia (NH_3_·H_2_O), and 2-propanol were provided by Xilong Chemical Co., Ltd. (Guangzhou, China); 3,3′,5,5′-Tetramethylbenzidine (TMB) was purchased from Tokyo Chemical Industry Co., Ltd. (Shanghai, China). Dulbecco’s modified Eagle’s medium (DMEM) and 1640 cell culture media were acquired from Thermo Fisher Scientific Inc. (Cleveland, OH, USA); 2-(N-morpholino)ethanesulfonic acid (MES) buffer (100 mM, pH 4.8), Cy3 dyes, and carboxylated AS14111 aptamer (from 5′ to 3′: HOOC-TTT TTT TTT GGT GGT GGT TGT GGT GGT GGT GG) were provided by Sangon Biotech Co., Ltd. (Shanghai, China). The human serum sample was kindly provided from a healthy volunteer, and all related experiments were approved by the Institutional Ethical Committee (IEC) of Guangxi Normal University.

### 2.2. Instrumentation

Transmission electron microscopy (TEM) images were acquired from a Talos 200S transmission electron microscope (Thermo Fisher Scientific Inc., Cleveland, OH, USA). Scanning electron microscope (SEM) and energy dispersive spectroscopy (EDS) were performed with a FEI Quanta 200 scanning electron microscope (Hitachi, Japan). Inductively coupled plasma mass spectrometry (ICP-MS) was conducted on Agilent 7700ce (Agilent, Santa Clara, CA, USA). Fourier transform infrared (FT-IR) spectrum was obtained using a Spectrum Two UATR FT-IR spectrometer (Perkin-Elmer, Waltham, MA, USA). Zeta potential was measured using a Nano ZS-90 Malvern Particle size potentiometer (Malvern Instruments, Worcestershire, UK). X-ray diffraction (XRD) patterns were collected on Rigaku D/max 2550 VB/PC X-ray diffractometer (Rigaku Company, Tokyo, Japan). Ultraviolet−visible (UV−vis) absorption spectra were collected on a Cary 60 spectrometer (Agilent, Santa Clara, CA, USA). Cell imaging was performed using a LSM 710 confocal laser scanning microscope (Zeiss, Jena, Germany).

### 2.3. Preparation of UIO-66(Fe/Zr)

The UIO-66(Fe/Zr) NPs were synthesized following a previously reported method [13]. In brief, 0.8 mM ZrCl_4_, 0.8 mM FeCl_3_·6H_2_O, and 0.8 mM H_2_BDC were mixed into 25 mL of DMF, followed by the addition of 5 mL of acetic acid. After ultrasonic treatment at ambient temperature, the mixture was transferred to a 50-mL Teflon-lined hydrothermal reactor and reacted at 120 °C for 12 h. Subsequently, the obtained products were washed with ultrapure water and ethanol three times, respectively. After drying the products at 60 °C overnight, UIO-66(Fe/Zr) powder was collected for further use. For the preparation of Cy3-dye-incorporated UIO-66(Fe/Zr), 9.7 mg of UIO-66(Fe/Zr) NPs were mixed with 0.03 mM Cy3 dyes, followed by stirring for 24 h.

### 2.4. Preparation of AS1411-Functionalized Fe_3_O_4_@SiO_2_

First, Fe_3_O_4_@SiO_2_ core-shell NPs were synthesized using a reported sol-gel strategy [14]. Typically, 0.15 g of Fe_3_O_4_ NPs were ultrasonically dispersed into a mixture solution containing 70 mL of ultrapure water, 280 mL of ethanol, and 5.0 mL of NH_3_·H_2_O (28 wt%), followed by a slow addition of 4.0 mL of TEOS. After stirring for 10 h, the resulting Fe_3_O_4_@SiO_2_ NPs were collected after magnetic separation and washing with ultrapure water and ethanol.

Then, 0.3 g of Fe_3_O_4_@SiO_2_ NPs were mixed with 303 mL of 2-propanol containing 3 mL of APTES. After stirring at 70 °C for 6 h, Fe_3_O_4_@SiO_2_-NH_2_ NPs were obtained by magnetic separation. Subsequently, the carboxylated aptamer AS1411 and Fe_3_O_4_@SiO_2_-NH_2_ (250 μL, 5 mg/mL) were added to 500 μL of MES (100 mM, pH 4.8) solution containing 9.6 mg of EDC. After incubation at 37 °C overnight, the obtained AS1411-functionalized Fe_3_O_4_@SiO_2_ (Fe_3_O_4_@SiO_2_-Apt) NPs were magnetically isolated and resuspended in Tris-HCl buffer (10 mM, pH 7.4) for future use.

### 2.5. Assessment of Peroxidase-Like Nanozyme Activity 

To investigate the peroxidase-like nanozyme activity, UIO-66(Fe/Zr) or Fe_3_O_4_@SiO_2_-Apt were added into 200 μL of NaAc-HAc buffer (10 mM, pH 3.5) containing TMB (50 μL, 10 mM) and H_2_O_2_ (50 μL, 20 mM). After reacting at 37 °C for 30 min, the catalytic reaction was terminated using 1 M H_2_SO_4_. The resulting solution was photographed and measured using an UV-vis spectrophotometer.

### 2.6. Cell Culture

Human cervical cancer HeLa cells were cultured in 1640 medium containing 10% FBS and 1% penicillin-streptomycin. For the cases of human hepatoma HepG2 cells and mouse fibroblast L929 cells, DMEM medium was used. All the cell lines were incubated in a humidified atmosphere of 5% CO_2_ at 37 °C.

### 2.7. Colorimetric Detection of Cancer Cells

First, HeLa cells with different concentrations (5 × 10^2^, 1 × 10^3^, 2 × 10^3^, 3 × 10^3^, 5 × 10^3^, 8 × 10^3^, 9 × 10^3^, 1 × 10^4^, 2 × 10^4^, 3 × 10^4^ cells/mL) were cultured in 1640 medium overnight. Then, fresh cell medium containing Fe_3_O_4_@SiO_2_-Apt was added and incubated for 2 h. After washing with PBS (10 mM, pH 7.4), the cells were digested and magnetically collected. Subsequently, UIO-66(Fe/Zr) were added into the resulting cells and incubated at 37 °C for 30 min. After removing free UIO-66(Fe/Zr), 200 μL of NaAc-HAc buffer (10 mM, pH 3.5) containing TMB (50 μL, 10 mM) and H_2_O_2_ (50 μL, 20 mM) was added and reacted at 37 °C for 30 min. After terminating the reaction using 1 M H_2_SO_4_, the absorbance spectra were collected by an UV-vis spectrophotometer. To investigate the potential in detecting real samples, HeLa cells with different concentrations (5 × 10^3^, 8 × 10^3^, 1 × 10^4^ cells/mL) were spiked into 1000-fold diluted human blood, respectively, followed by colorimetric analysis.

## 3. Results and Discussion

### 3.1. Principle of Colorimetric Sandwich-Type Detection of Cancer Cells

**Figure 1:**
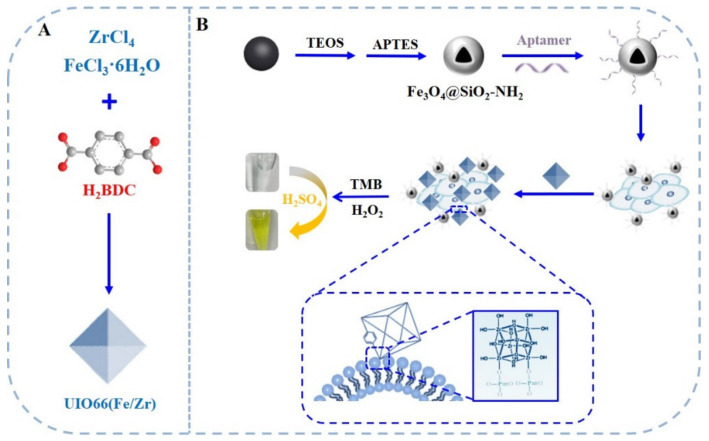
Schematic illustration of (**A**) UIO-66(Fe/Zr) synthesis and (**B**) their application in constructing a single aptamer-dependent sandwich-type biosensor for colorimetric detection of cancer cells.

As shown in Figure 1A, UIO-66(Fe/Zr) NPs were synthesized by a one-step hydrothermal method using ZrCl_4_ and FeCl_3_·6H_2_O as metal precursors and H_2_BDC as organic ligands. In order to guarantee selective detection of target cancer cells, a novel sandwich-type aptasensor was proposed using aptamer-functionalized Fe_3_O_4_@SiO_2_ core-shell NPs to selectively capture and magnetically separate cancer cells from complex biological systems (Figure 1B). Considering bare Fe_3_O_4_ NPs also own peroxide-like nanozyme activity, Fe_3_O_4_@SiO_2_ core-shell NPs originating from the silylanization of Fe_3_O_4_ NPs were adopted here to improve the signal-to-noise ratio toward cancer cell analysis. Taking advantage of the Zr-O-P coordination bond between phosphate units in the phospholipid bilayer of the cell membrane and zirconium nodes on MOFs, UIO-66(Fe/Zr) could directly bind to the cell surface. In addition, UIO-66(Fe/Zr) possesses a strong peroxidase-like nanozyme activity, which can catalyze H_2_O_2_-mediated oxidation of colorless TMB to form yellow-colored oxidized ones (oxTMB) with the aid of 1 M H_2_SO_4_, providing an excellent alternative for colorimetric analysis of cancer cells.

### 3.2. Characterization of UIO-66(Fe/Zr) and Fe_3_O_4_@SiO_2_-Apt

**Figure 2:**
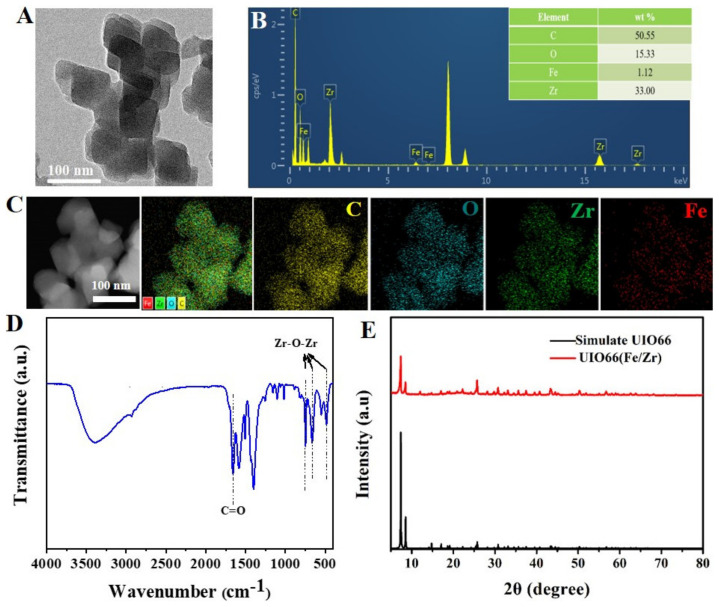
(**A**) Transmission electron microscopy (TEM) image, (**B**) elemental analysis, (**C**) EDS images, and (**D**) FT-IR spectrum of the prepared UIO-66(Fe/Zr). The inset shows the corresponding percentage of elements C, O, Fe, and Zr. (**E**) XRD patterns of the prepared UIO-66(Fe/Zr) and simulate UIO-66.

After a simple hydrothermal process, UIO-66(Fe/Zr) NPs were successfully prepared with a size ranging from 70 nm to 96 nm (Figure 2A). Observed from the EDS (Figure 2B) and elemental imaging (Figure 2C), the elements of C, O, Zr, and Fe were uniformly distributed among the bimetallic MOFs. Furthermore, the molar ratio of Fe and Zr was calculated to be 1:32 according to the ICP-MS analysis. Observed from the FT-IR (Figure 2D), the stretching vibration of the C=O bond in the H_2_BDC organic ligand displayed a characteristic absorption peak at 1660 cm^−1^ [15], while the vibration of Zr-O-Zr yielded triple peaks at 766, 662, 482 cm^−1^ [16]. Powder XRD results demonstrated the prepared UIO-66(Fe/Zr) exhibited a similar crystal structure to that of simulated UIO-66, as evidenced by the characteristic diffraction peaks at 7.36°, 8.48°, and 25.72° assigned to the (111), (002), and (224) crystal planes (CCDC-1507786), respectively (Figure 2E) [17]. These results indicated that the introduction of additional Fe nodes was unable to disturb the intact metal–organic framework of UIO-66(Zr), consistent with that reported previously [13]. Taken all the above results into consideration, it can be concluded that a part of the Zr nodes in the UIO-66 framework were carefully displaced by Fe nodes, and the similar Fe-O-Fe bond to Zr-O-Zr bond ensured a comparable spatial configuration of the UIO-66(Fe/Zr) to that of UIO-66(Zr).

Next, Fe_3_O_4_@SiO_2_ core-shell NPs were prepared via sonication-assisted coating of a silica shell on Fe_3_O_4_ NPs core. As shown in Figure S1, spherical Fe_3_O_4_ NPs had a mean diameter of approximately 320 nm. After being coated with a thin silica shell (approximately 32 nm), an obvious core-shell structure was observed for the obtained Fe_3_O_4_@SiO_2_ NPs. Strong asymmetric stretching peaks of the Si-O-Si bond at 1207 and 1079 cm^−1^ appeared [18], accompanied by the disappearance of the characteristic peak of the Fe-O-Fe bond at 593 cm^−1^ [19], which indicated the successful coating of SiO_2_ on the surface of Fe_3_O_4_ NPs (Figure S2A). For the formed Fe_3_O_4_@SiO_2_ core-shell NPs, aminated treatment gave rise to a positive zeta potential (Figure S2B). However, further functional modification with aptamer AS1411 molecules induced the resultant Fe_3_O_4_@SiO_2_-Apt NPs to be negative-charged.

### 3.3. Assessment of Peroxidase-Like Nanozyme Activity 

**Figure 3:**
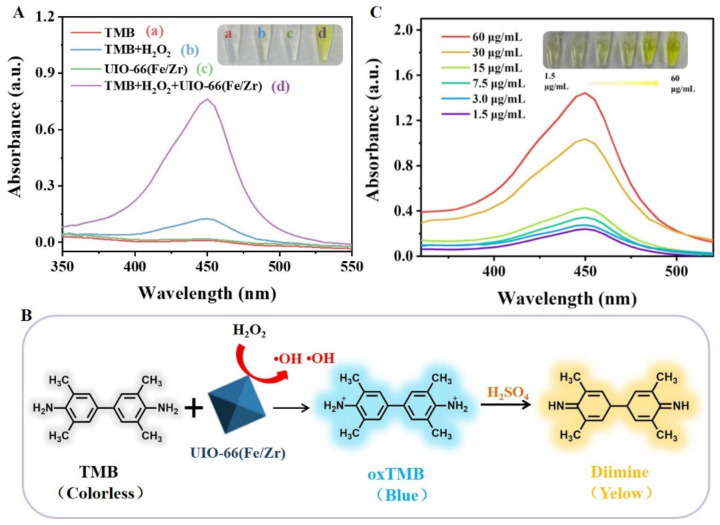
(**A**) UV-vis absorbance spectra of different solution. The insets showing the images of their corresponding solution. (**B**) Schematic illustration of UIO-66(Fe/Zr)-mediated catalytic oxidation of TMB-H_2_O_2_ system. (**C**) UIO-66(Fe/Zr) concentration-dependent UV-vis absorbance spectra of TMB-H_2_O_2_ system. The insets show the images of their corresponding solution.

The peroxidase-like nanozyme activity was visually evaluated using the typical TMB-H_2_O_2_ colorimetric system [7]. As shown in Figure 3A, under the condition of the acetate buffer, the mixture of TMB and H_2_O_2_ almost remained colorless with a low absorption peak intensity at 450 nm, indicating a relatively slow reaction efficiency (inset b). After introduction of UIO-66(Fe/Zr) (inset d), the solution color of the TMB+H_2_O_2_ system turned to be yellow. Since UIO-66(Fe/Zr) NPs alone exhibited negligible absorption at 450 nm (inset c), the above-mentioned yellow solution was, thus, attributed to the catalytic decomposition of H_2_O_2_ by UIO-66(Fe/Zr) NPs to promote the oxidation of TMB (Figure 3B). Furthermore, the absorption peak intensity was positively related to the added amount of UIO-66(Fe/Zr) NPs (Figure 3C), revealing the feasibility of visual analysis using a UIO-66(Fe/Zr)-mediated colorimetric system.

In order to obtain a low background signal, Fe_3_O_4_@SiO_2_-Apt were expected without peroxidase-like activity. As anticipated, bare Fe_3_O_4_ NPs did catalyze the decomposition of hydrogen peroxide to accelerate TMB oxidization (inset b, Figure S3). For the case of Fe_3_O_4_@SiO_2_-Apt, effective coverage of Fe_3_O_4_ with a thin shell of SiO_2_ resulted in a significant decrease in the number of catalytic sites, and the peroxidase-like activity was almost lost (insets c and d, Figure S3).

### 3.4. Construction of a Single-Aptamer-Based Sandwich-Type Biosensor

**Figure 4:**
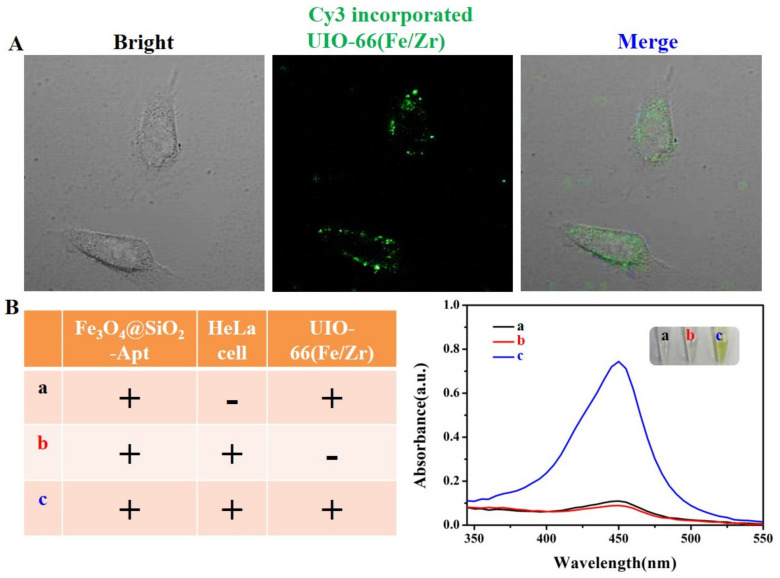
(**A**) Bright-field image, fluorescence image, and overlay of the fluorescence and bright-field images of HeLa cells after incubation with bare Cy3 dyes-incorporated UIO-66(Fe/Zr) NPs. (**B**) UV-vis absorbance spectra of TMB-H_2_O_2_ system after different treatments. The insets show the images of their corresponding solution.

The direct binding of bare UIO-66(Fe/Zr) to target cancer cells was of vital importance for constructing a single-aptamer-based sandwich-type biosensor. To this end, Cy3-dyes-incorporated UIO-66(Fe/Zr) NPs were prepared for fluorescence-assisted positioning of their binding sites (Figure S4). As a proof-of-concept assay, we attempted to incubate human cervical cancer HeLa cells with Cy3-dyes-incorporated UIO-66(Fe/Zr) NPs. As imaged by confocal laser scanning microscopy (CLSM), the outline of the HeLa cell was clearly lighted up, taking advantage of the luminance of Cy3 (Figure 4A), which proved the successful binding of bare UIO-66(Fe/Zr) NPs to the surface of HeLa cells. The resultant HeLa cell maintained the original morphology, which demonstrates the excellent biocompatibility of UIO-66(Fe/Zr) NPs.

Inspired by the direct binding of bare UIO-66(Fe/Zr) NPs to HeLa cells, a colorimetric sandwich-type aptasensor was then designed using Fe_3_O_4_@SiO_2_-Apt to selectively capture and magnetically separate HeLa cells through specific recognition of aptamer AS1411 to the overexpressed nucleolin [20,21]. To achieve the colorimetric detection of HeLa cells, Fe_3_O_4_@SiO_2_-Apt were first incubated with HeLa cells at 37 °C for 2 h, followed by the addition of UIO-66(Fe/Zr) NPs and incubation at 37 °C for another 30 min. After magnetic separation of the free nanozymes, the formed sandwich-type structure was added into the TMB+H_2_O_2_ system. Without HeLa cells or UIO-66(Fe/Zr), the solution color of TMB+H_2_O_2_ system in the presence of Fe_3_O_4_@SiO_2_-Apt was nearly colorless, and weak absorption at 450 nm was generated (curves a and b, Figure 4B). For the colorimetric system treated with a stable sandwich-type structure (curve c, Figure 4B), the yellow solution appeared with an over 7-fold absorbance intensity compared to that of the blank sample (inset c, Figure 4B), which demonstrates the feasibility of colorimetric detection of HeLa cells.

### 3.5. Colorimetric Detection of Cancer Cells

**Figure 5:**
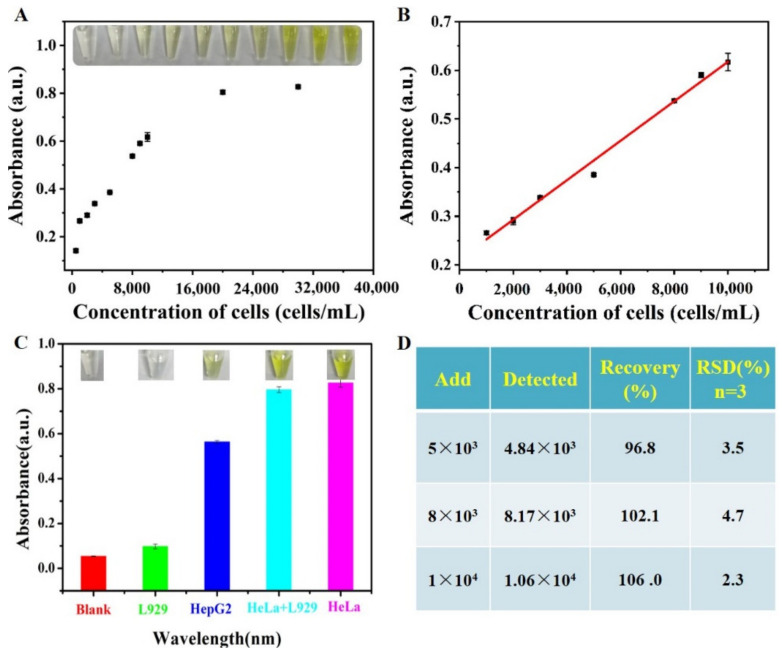
(**A**) The absorbance intensity response of TMB-H_2_O_2_ system at 450 nm toward various concentrations of HeLa cells (5 × 10^2^, 1 × 10^3^, 2 × 10^3^, 3 × 10^3^, 5 × 10^3^, 8 × 10^3^, 9 × 10^3^, 1 × 10^4^, 2 × 10^4^, 3 × 10^4^ cells/mL). The insets show the images of their corresponding solution. (**B**) Linear relationship of the absorbance intensity versus HeLa cell concentration from 10^3^ to 10^4^ cells/mL. (**C**) Specificity of the colorimetric sandwich-type aptasensor toward different interference cells. The insets show the images of their corresponding solution. (**D**) The recovery assays.

As the concentration of HeLa cells increased from 0 to 2 × 10^4^ cells/mL, the colorimetric system gradually darkened in solution color (the inset, Figure 5A). By measuring the absorption intensity at 450 nm, quantitative analysis further confirmed a positive correlation between HeLa cell concentration and the oxidation efficiency of TMB (Figure 5A). These results were originated from the fact that the presence of more HeLa cells meant more cell membrane-bound UIO-66(Fe/Zr) nanozymes, resulting in a higher catalytic reaction dynamic of TMB oxidation by H_2_O_2_. As illustrated in Figure 5B, a good linear relationship was attained between the absorption intensity at 450 nm and the cell concentration ranging from 10^3^ to 10^4^ cells/mL. The detection limit (LOD) was calculated to be 481 cells/mL based on 3σ/k (σ and k representing the standard deviation of blank signal and the curve slope, respectively).

To further verify the sensing selectivity toward HeLa cells, a same concentration of human hepatoma HepG2 cells and mouse fibroblast L929 cells were tested with the proposed colorimetric sandwich-type aptasensor, respectively. When compared with the blank sample, the group of L929 cells caused no apparent color change, while a significant yellow solution was produced for the samples of HepG2 and HeLa cells (Figure 5C). Higher response sensitivity toward cancer cells might be ascribed to a higher expression level of nucleolin on cancer cells than that on normal cells [8], which verifies the sensing specificity of the colorimetric sandwich-type aptasensor. Furthermore, this aptasensor could accurately distinguish HeLa cells from an analogue biological environment via mixing HeLa and L929 cells (light-blue column, Figure 5C).

Encouraged by the excellent selectivity, the developed aptasensor was employed for the analysis of real samples by spiking different concentrations of HeLa cells into 1000-fold diluted healthy human blood. A satisfactory recovery rate between 96.8% and 106.0% with an acceptable relative standard deviation (RSD) value was obtained (Figure 5D), which fully demonstrated the accuracy and reliability of the developed aptasensor in analyzing target cancer cells in the complex biological system.

## 4. Conclusions

In summary, a novel single aptamer-dependent sandwich-type biosensor was proposed for simple, sensitive, and specific detection of HeLa cells using Fe_3_O_4_@SiO_2_-Apt to selectively capture and magnetically separate target cancer cells, and using UIO-66(Fe/Zr) NPs to output amplified colorimetric signals. Through designing appropriate metal nodes in the given MOFs, the prepared UIO-66(Fe/Zr) NPs show desirable integrated properties, that is, the intrinsic peroxidase-like activity originated from Fe nodes, and direct binding to the cell surface using the Zr-O-P coordination bond between phosphate units in the phospholipid bilayer of the cell membrane and zirconium nodes. These unique properties ensured high sensing sensitivity and broke through the limitation of the requirement of labeling two antibodies or aptamers. The constructed colorimetric aptasensor could achieve visual detection of HeLa cells in the range of 10^3^-10^4^ cells/mL with a detection limit of 481 cells/mL. Such a novel single aptamer-dependent colorimetric sandwich-type biosensor has great potential in the diagnosis and treatment evaluation of cancer.

## Data Availability

The data presented in this study are available within the article and its Supplementary Materials. Other data that support the findings of this study are available upon request from the corresponding author.

## Supplementary Materials

The following supporting information can be downloaded at: https://www.mdpi.com/article/10.3390/bios13020225/s1, Figure S1: TEM images of Fe_3_O_4_ and Fe_3_O_4_@SiO_2_ NPs; Figure S2. (A) FT-IR spectra of Fe_3_O_4_, Fe_3_O_4_@SiO_2_, Fe_3_O_4_@SiO_2_-NH_2_. (B) Zeta potential of Fe_3_O_4_@SiO_2_, Fe_3_O_4_@SiO_2_-NH_2_, Fe_3_O_4_@SiO_2_-Apt; Figure S3. UV-vis absorbance spectra of the TMB-H_2_O_2_ system after different treatments; Figure S4. Fluorescence emission spectra of UIO-66(Fe/Zr) before and after incubation with Cy3 dyes.
