# Supplementary material for: A Single Aptamer-Dependent Sandwich-Type Biosensor for the Colorimetric Detection of Cancer Cells via Direct Coordinately Binding of Bare Bimetallic Metal–Organic Framework-Based Nanozymes
Source: Biosensors (Basel). 2023 Feb 3;13(2):225. doi: 10.3390/bios13020225 (PMC9954218; doi:10.3390/bios13020225)
Supplement: Supplementary file 1 [file biosensors-13-00225-s001.zip › biosensors-2193734-supplementary.pdf]

Supporting information

A Single Aptamer-Dependent Sandwich-Type Biosensor for the Colorimetric Detection of Cancer Cells *Via* Direct Coordinately Binding of Bare Bimetallic Metal–Organic Framework-based Nanozymes

Yuhui Zhu,[a,†] Xueting Fang,[a,†] Xiaofei Lv,[a] Meijun Lu,[a] Hui Xu,[b] Shengqiang Hu,[a] Shulin Zhao and Fanggui Ye[a*]

[a] State Key Laboratory for the Chemistry and Molecular Engineering of Medicinal Resources, School of Chemistry and Pharmaceutical Sciences, Guangxi Normal University, Guilin 541004, P. R. China
[b] Nanxian Inspection and Testing Center of Yiyang City in Hunan Province, Yiyang 413299, China
* Correspondence: fangguiye@163.com (F.Y.)
† Y.Z. and X.F. contributed equally to this work.

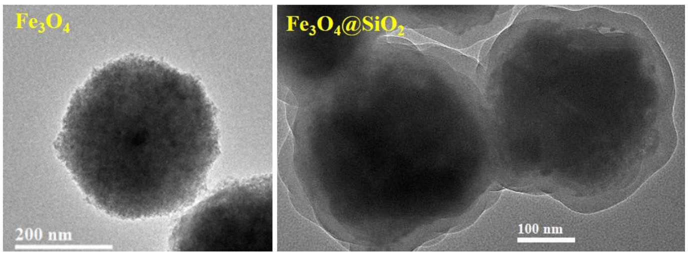

Figure S1. TEM images of Fe_3O_4 and $Fe_3O_4@SiO_2$.

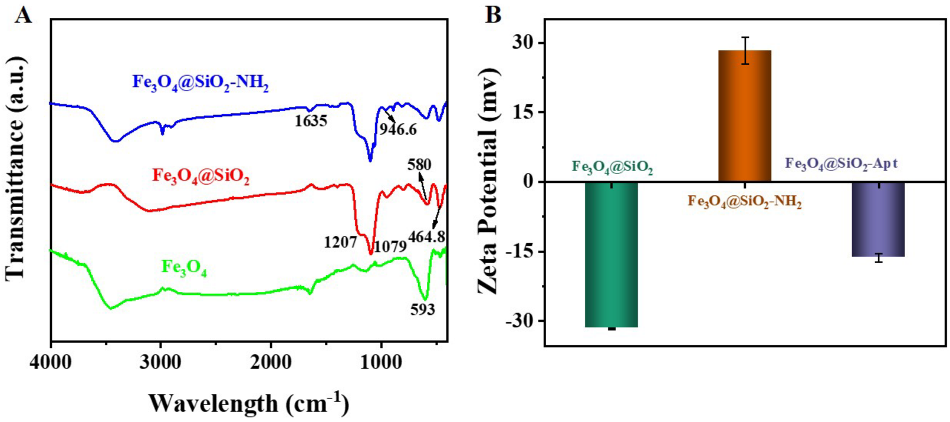

Figure S2. (A) FT-IR spectra of Fe_3O_4, $Fe_3O_4@SiO_2$, $Fe_3O_4@SiO_2$-NH_2. (B) Zeta potential of $Fe_3O_4@SiO_2$, $Fe_3O_4@SiO_2$-NH_2, $Fe_3O_4@SiO_2$-Apt.

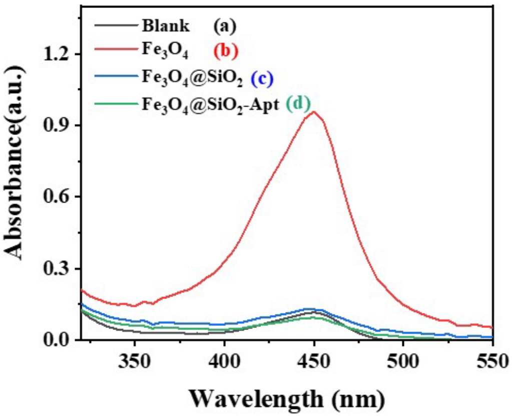

Figure S3. UV-vis absorbance spectra of the TMB-H_2O_2 system after different treatments.

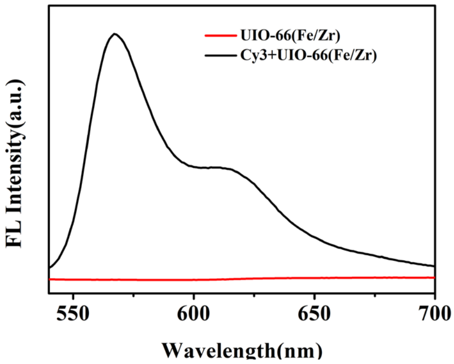

Figure S4. Fluorescence emission spectra of UIO-66(Fe/Zr) before and after incubation with Cy3 dyes.
